# Cancer advocacy, barriers and associated factors among adolescents and young adults with cancer in Africa

**DOI:** 10.3332/ecancer.2025.1888

**Published:** 2025-04-10

**Authors:** Chinomso Ugochukwu Nwozichi, Omolabake Salako, Anita Frimpomaa Oppong, Margaret Olutosin Ojewale

**Affiliations:** 1 Wellstar School of Nursing, Kennesaw State University, Kennesaw, GA 30101, USA; 2 Department of Nursing Research, Nursology Academy, Ilishan Remo, Ogun State, 121103, Nigeria; 3 School of Nursing, University of Connecticut, Storrs, CT 06269, USA; 4 Nigerian Army College of Nursing, Yaba, Lagos 101245, Nigeria

**Keywords:** cancer, advocacy, Africa, adolescent and young adult

## Abstract

Adolescents and young adults (AYAs) are at a critical juncture in their lives, often pursuing education, starting careers, forming relationships and gaining independence. Cancer disrupts these pivotal life transitions, causing significant emotional and psychological stress. Advocacy plays a crucial role in promoting awareness, access to care and support for AYAs with cancer. This paper aims to identify and examine the potential barriers to access to cancer treatment and advocacy among AYAs with cancer in Africa. A selective literature review was conducted to provide a foundation for the discussion. The search involved databases such as PubMed, Scopus and Google Scholar, using keywords such as ‘AYAs,’ ‘cancer,’ ‘advocacy,’ ‘Africa,’ ‘barriers’ and ‘factors.’ Relevant articles were selected based on their relevance, recency and quality, focusing on empirical studies and seminal and theoretical papers. Drawing from existing literature, expert opinions and case studies, several key barriers are explored, including social and cultural stigma surrounding cancer, limited access to health education and support services, economic constraints and inadequate healthcare infrastructure. Sociocultural norms and taboos can lead to a lack of open dialogue about cancer, hindering advocacy efforts. Furthermore, poverty and financial constraints often impede access to treatment and support resources, limiting opportunities for AYAs to engage in advocacy initiatives. Inadequate healthcare systems, lack of specialised care facilities and limited training for healthcare professionals also pose significant challenges. This paper highlights the urgent need to address these multifaceted barriers through collaborative efforts involving healthcare providers, policymakers and community organisations. Recommendations are provided for developing culturally sensitive advocacy programs, improving health literacy and strengthening healthcare infrastructure to empower AYAs with cancer and amplify their voices in Africa.

## Introduction

Receiving a cancer diagnosis when someone is an adolescent or young adult disrupts the normal course of their development. This critical life phase involves significant physical, social and emotional growth and maturation [7]. However, cancer and its treatment can substantially impede the progression of these essential developmental milestones and processes [60]. In the face of a cancer diagnosis, the fight for better health becomes a collective effort, inclusive of patient advocacy by healthcare professionals and self-advocacy by cancer patients themselves. Adolescents and young adults (AYAs) are frequently diagnosed at later stages due to a lack of awareness and specific screening programs for this age group in Africa [49]. They often fall into a gap between pediatric and adult oncology services, leading to delayed diagnoses and suboptimal treatment protocols that are not tailored to their unique needs [20].

AYAs with cancer generally occupy a unique and often overlooked demographic in the oncology landscape. Moreover, historically, AYAs with cancer have been treated in either pediatric or adult healthcare facilities, often divided by an 18-year age limit [20]. This separation has led to suboptimal outcomes for AYAs compared to pediatric and adult patients. In recent years, the oncology research community has become increasingly aware that this strict separation between pediatric and adult care is inadequate, resulting in several shortcomings for AYA cancer patients compared to their younger and older counterparts [73]. The field of AYA oncology has emerged to address these specific needs, and there is a consensus on the necessity for specialised AYA oncology units and specialists [59]. Despite some improvements in survival rates for certain cancer types among AYAs in Africa, these improvements are not as significant as those observed in Europe and the United States [20].

In many African countries, AYAs with cancer must navigate this challenging reality while confronting limited resources and strained healthcare systems [57]. These young individuals are tasked with negotiating the transition between childhood and adulthood while simultaneously dealing with a life-threatening illness [63]. Given these unique challenges, there is a growing recognition of the need for AYAs to take an active role in their care and shape the broader landscape of cancer treatment and support. This active participation often takes the form of advocacy. Advocacy in cancer care extends beyond individual treatment plans, transforming patients into active participants and shaping the landscape of their well-being. For AYAs in Africa, this could involve advocating for inclusion in clinical trials and researching new and innovative treatment approaches for their specific cancers. Access to clinical trials, which AYAs in Africa do not readily have, is a critical component of cancer advocacy for AYAs, offering potential improvements in treatment and survival, contributing to scientific advancements and empowering patients and families [34].

Moreover, advocacy allows AYAs to challenge the stigma and misinformation surrounding cancer, particularly prevalent in some African communities [53]. By raising their voices, they can advocate for educational programs that dispel myths and encourage early detection, a crucial factor in improving cancer outcomes. Promoting health advocacy for adolescents and young people with cancer requires skills such as empathy and listening, as well as exploring opportunities where the patient’s voice is most important [42]. Ultimately, by breaking barriers, AYAs in Africa possess immense potential to become powerful advocates, not just for themselves but for future generations facing cancer. Their lived experiences hold invaluable insights that can inform more effective and youth-centered cancer care strategies across Africa.

This paper aims to discuss the potential barriers to barriers to access to cancer treatment and advocacy among AYAs with cancer in Africa, drawing from existing literature. A selective literature review was conducted to provide a foundation for the discussion. The search involved databases such as PubMed, Scopus and Google Scholar, using keywords like ‘AYAs,’ ‘cancer,’ ‘advocacy,’ ‘Africa,’ ‘barriers’ and ‘factors.’ Relevant articles were selected based on their relevance, recency and quality, focusing on empirical studies and seminal and theoretical papers.

### What is advocacy?

One-quarter of the world’s population is aged 10–24 years, as defined by the World Health Organisation as AYAs. Despite representing a significant proportion of the global population, this age group is often overlooked in global sustainable care practices such as patient and self-advocacy [58]. A cancer diagnosis for an adolescent or young adult in Africa can also be a catalyst for remarkable transformation, both for the individual and the broader community. This transformation is often seen through the lens of personal growth, societal impact and the drive to advocate for better healthcare systems. Hypothetically, consider a young woman named Amina from a rural village in Nigeria. At the age of 23, she is diagnosed with breast cancer, a shocking revelation that initially plunges her into despair. However, as she navigates her treatment journey, Amina discovers an inner strength she never knew she had. The rigorous regimen of chemotherapy, surgeries and hospital visits tests her resilience, but it also ignites a newfound determination to survive and thrive. Amina’s journey is marked by profound personal growth. She learns to advocate for herself, push for the best possible care and educate herself about her condition. The support from her family and community becomes a lifeline, strengthening her bonds with loved ones. Through this adversity, Amina emerges more resilient and more appreciative of life’s small joys. Her battle with cancer transforms her outlook on life, instilling in her a sense of purpose and a desire to give back. Inspired by Amina’s courage, the community comes together to support other cancer patients. They organise fundraising events to help cover medical costs for those in need, fostering a spirit of solidarity and collective responsibility. Amina becomes a local hero, her story a beacon of hope and a reminder that cancer, though formidable, can be faced head-on with the proper support and determination. Beyond her immediate community, Amina’s experience propels her into a larger role as an advocate for better cancer care in Africa. She connects with national and international cancer organisations, sharing her insights and lobbying for improvements in the healthcare system. Her advocacy efforts draw attention to the critical need for accessible cancer treatments, better diagnostic facilities and comprehensive support services for AYAs.

Amina’s voice adds to the growing chorus calling for policy changes and increased funding for cancer research and care. Her story underscores the urgent need for specialised training for healthcare professionals, ensuring they can address the unique needs of AYA cancer patients. Her advocacy leads to the establishment of a local support group for young cancer patients, providing a safe space for sharing experiences and fostering mutual support. Amina’s transformation from a cancer patient to a community leader and advocate exemplifies the profound impact a cancer diagnosis can have on an AYA in Africa. Her personal growth, driven by the resilience and the will to survive, sparks broader societal changes, from community mobilisation to systemic healthcare improvements.

The concept of patient advocacy covers protection from incompetency or misconduct in healthcare; provision of information about the patient’s diagnosis, treatment and prognosis; maintaining self-control, individualisation and humanity; and the identification and correction of inequalities in the delivery of health services [1]. Advocacy empowers individual patients to become agents of change. It is about ensuring access to specialised care tailored to their unique needs, which might differ significantly from those of adults or children. Health care transition from pediatric to adult-focused services is a longitudinal process driven by the collaboration and interactions between AYA patients, their families, providers, healthcare agencies and the environment [62]. Therefore, advocacy transcends simply speaking up for individual needs; it is about harnessing the power of one’s story to create positive change for a larger community.

Self-advocacy can be defined as the ability of an individual with cancer to overcome challenges in getting their preferences, needs and values met [75]. For instance, Amina’s story, shared through local media platforms or patient advocacy groups, can inspire others to speak up, demand better resources and be resilient throughout their journey. Self-advocacy training in building self-awareness, communication skills and leadership can lead to the promotion of healthcare outcomes for adolescents and young people with cancer in Africa [4]. Advocacy empowers AYAs to move beyond the limitations of their diagnosis and become changemakers. This could involve lobbying governments to allocate resources for dedicated cancer units within hospitals that cater specifically to the needs of AYAs, ensuring age-appropriate treatment protocols and psychosocial support. They can advocate for policy changes prioritising funding for AYA oncology programs in Africa. AYAs who have cancer have leveraged the Internet to gain a better understanding of their disease and connect across geographic boundaries with others facing the same challenges [6]. By raising their voices, AYAs can influence research agendas, ensuring a focus on cancers that disproportionately affect young people in Africa. This journey from patient to changemaker is a powerful testament to the resilience and leadership potential of AYAs in Africa. Through advocacy, they can transform their experiences into a force for a more equitable and effective cancer care system across the continent.

### The impact of advocacy on AYAs with cancer

AYAs are at a critical juncture in their lives, often pursuing education, starting careers, forming relationships and gaining independence. Cancer disrupts these pivotal life transitions, causing significant emotional and psychological stress. A cancer diagnosis throws a curveball at anyone’s life, but for AYAs in Africa, the challenges are often amplified. Beyond the physical fight against the disease, AYAs in Africa often face unique hurdles – limited access to specialised care, a dearth of research focused on their age group and the isolating experience of navigating illness during a crucial developmental stage. AYAs diagnosed with cancer in Africa face unique challenges, particularly when it comes to reliance on their parents for care [28]. The dependency on family, especially parents, is exacerbated by financial constraints and the lack of comprehensive health insurance. This dynamic introduces a complex set of issues that impact the well-being and treatment outcomes for AYAs. African parents, already stretched thin by daily living expenses and supporting other siblings, now face the monumental task of funding an AYA’s cancer treatment [55]. However, amidst these challenges lies a powerful tool: advocacy.

Self-advocacy allows individuals, especially AYAs with cancer, to overcome challenges related to their health, care and well-being [74]. Raising their voices and advocating for their needs can have a profound impact on AYAs with cancer in Africa. This impact goes beyond just individual needs. Patients advocating for their needs and priorities is greatly beneficial to their health, especially with increasing emphasis on patient self-management [79]. Effective advocacy by AYAs can create a ripple effect, leading to improved access to treatment and resources, amplifying research efforts focused on cancers that specifically affect young people, and fostering a strong network of peer support and community building. The skill of self-advocacy among AYAs with cancer changes the focus of their cancer care to what is important to them and promotes optimised patient-centered care [5]. Let us explore these three key areas where advocacy empowers AYAs with cancer in Africa to take control of their health journey and advocate for a brighter future.

## Improved access to treatment and resources

For AYAs with cancer in Africa, the fight for better health often starts with a fight for access. Rural counties across Africa have high rates of cancer-related mortality and other negative treatment outcomes, due to low access to healthcare [43]. Limited resources and overwhelmed healthcare systems can create significant barriers to obtaining specialised care tailored to their unique needs. Limited access to timely diagnosis, affordable, effective treatment and high‐quality care are just some of the factors that lead to disparities in cancer survival, especially for AYAs in Africa [17]. However, advocacy empowers AYAs to become vocal advocates for themselves and their peers. By raising their voices, they can demand increased access to essential diagnostic tools and ensure early detection of cancers, crucial for improving survival rates. Enabling AYA cancer patients to manage the medical and emotional consequences due to cancer care and treatment is crucial to optimising health and recovery across the continuum of cancer [32]. When individuals speak for themselves, they draw from their own experiences and emotions, which brings an undeniable authenticity to their message. Imagine a young cancer patient or survivor addressing a crowd about the challenges of undergoing treatment. The raw emotion and genuine perspective conveyed through their words resonate deeply with the audience. This authenticity makes the message more credible and impactful than if a representative were to relay the same information on their behalf. The power of self-expression lies in its authenticity, credibility and emotional resonance. When people speak for themselves, their messages carry a weight and sincerity that are difficult to achieve through a third party. This direct communication fosters personal connections, builds trust and highlights the individual’s agency and integrity, making their voice a powerful tool for advocacy and change. This transformation highlights the potential for individual stories to drive significant social change, demonstrating that there is the capacity for growth, impact and hope even in the face of profound adversity.

Implementing evidence‐based interventions through advocacy for AYAs could substantially reduce cancer disparities [35]. Moreover, advocacy allows AYAs to champion increased access to vital support services, such as psychological counseling, nutritional guidance and fertility preservation options. These holistic approaches address the emotional and physical toll of cancer, improving overall well-being and empowering AYAs to focus on their recovery journey. Through advocacy, survival outcomes improved for young adults with cancer following the expansion of health insurance coverage which increased access to cancer care and treatment [67].

One significant outcome of effective advocacy could be the development of dedicated AYA oncology units within African hospitals. These units would provide age-appropriate treatment protocols, address psychosocial concerns specific to AYAs, like navigating school or work during treatment, and offer a more supportive environment compared to adult-centered wards. While countries such as Canada [70], the United States of America [65, 82], and Italy [21] have developed dedicated cancer units for AYAs, this model, or at least establishing an AYA multidisciplinary team, could improve treatment outcomes for AYAs with cancer in Africa.

However, it is important to acknowledge the resource constraints in many African healthcare systems. While establishing fully dedicated AYA oncology units may be resource-intensive for many African countries, a stepped approach could be considered. This might involve initially creating AYA-focused multidisciplinary teams within existing oncology departments, gradually implementing age-appropriate protocols and introducing targeted psychosocial support services. As resources allow, hospitals could then work towards developing more comprehensive AYA-specific spaces and programs. Additionally, regional collaborations and partnerships with international organisations could help support the development of these specialised services in a cost-effective manner. Ultimately, effective advocacy translates into a more equitable healthcare landscape where AYAs in Africa have the same opportunities to access quality treatment and resources as their counterparts elsewhere.

## Amplifying research efforts focused on AYAs

Cancer research plays a critical role in developing new and effective treatments. However, research agendas often need to pay more attention to the specific needs of AYAs, particularly in Africa. Numerous barriers and facilitators of clinical trial enrolment include those that are system level and patient level, which can be mitigated by increased advocacy by adolescent and young cancer patients [41]. By sharing their experiences and highlighting the unique challenges they face, AYAs can influence research priorities to ensure a focus on developing therapies with minimal long-term side effects, crucial for AYAs who have a longer life expectancy compared to adults diagnosed with cancer.

Survival rates for AYAs aged 15 to 39 with cancer have not improved as significantly as those for pediatric and older adult cancer patients. This discrepancy is partly attributed to the lower participation of AYAs in clinical trials [39]. The significant challenges to adequate research include a lack of understanding of AYA cancer biology, access to specialised centers and available clinical trials– especially in African regions [24]. Researchers in developing countries have investigated the biological and genetic factors contributing to cancer in AYAs. Advocacy should trigger research investigating the genetic and molecular characteristics of cancers commonly affecting AYAs in Africa. However, advocacy can lead to increased funding for research dedicated to understanding the biological and social determinants of AYA cancers in Africa. There should be research on developing and implementing integrated care models that bring together oncologists, psychologists, social workers and other specialists to provide comprehensive care for AYAs in Africa. This can pave the way for developing more effective treatment protocols and supportive care programs tailored to the specific needs of this age group. Progress in reducing cancer morbidity and mortality among AYAs could be facilitated through more equitable access to health, increasing clinical trial enrollment and expanding research, especially across Africa [48]. This allows them to access potentially life-saving therapies and contributes valuable data that informs future research and drug development specifically tailored to AYA cancers. Understanding the cancer landscape specific to AYAs in Africa can help develop targeted prevention and treatment strategies. By amplifying research efforts focused on AYAs, advocacy empowers them to shape the future of cancer treatment in Africa, ensuring more effective and targeted therapies for future generations.

## Fostering peer support and community building among AYAs with cancer

A cancer diagnosis can be isolating, especially for AYAs in Africa, navigating a complex healthcare system and social stigma [50,54]. However, advocacy empowers AYAs to build a strong network of support. Peer support can reduce feelings of isolation and provide emotional and practical support from individuals who understand their experiences. Advocacy groups are a crucial part of the cancer support for AYAs, as psychological support for patients is considered an essential part of cancer survivorship, the significance of peer-to-peer support and rooted in principles of patient empowerment [64]. Through advocacy groups and online communities, AYAs can connect with peers facing similar challenges. A significant positive impact of shared personal experiences offers a greater understanding of the benefits of online peer-to-peer support for mental health and wellbeing, including for AYAs with cancer [61]. Sharing experiences, offering emotional support and exchanging practical information can provide a sense of belonging and empower AYAs to feel less alone.

Advocacy efforts can also lead to the development of mentorship programs connecting young patients with AYA cancer survivors. AYAs with cancer have identified connection to online communities of same-age peers to be essential for psycho-social support during cancer treatment [29]. These mentors can offer invaluable guidance and inspiration, demonstrating that a fulfilling life after cancer is possible. Furthermore, advocacy can lead to the creation of youth-friendly resources specifically designed to address the concerns of AYAs with cancer in Africa. Cancer advocay groups provide patients, especially AYAs with information, emotional relief and may promote empowerment [81]. These resources could include culturally sensitive educational materials, online support groups and workshops on coping with the emotional and social challenges of cancer. Frequent use of online advocacy groups was associated with less loneliness among AYA cancer patients [40]. Ultimately, effective advocacy fosters a sense of community and belonging for AYAs with cancer in Africa, providing them with the vital support network they need to navigate their journey and thrive.

### A spotlight of some advocacy groups in Africa

AYAs are a unique population: they are a diverse group between the ages of 15–39 years with distinct needs and experience numerous developmental milestones during this age range [8]. For AYAs with cancer in Africa, advocacy groups are more than just support networks;

they are catalysts for empowerment. AYAs affected by cancer constitute a vulnerable group in need of special support in pursuing everyday life as young people [11]. Advocacy groups provide a safe space for AYAs to voice their concerns, ask questions and connect with peers who understand their unique challenges. AYA with cancer have inequitable access to oncology services, most especially expert cancer care that focus on their unique needs [24]. Empowered with these tools, AYAs can confidently engage with healthcare professionals, policymakers and the broader community, advocating for their needs and the needs of others facing similar challenges.

## Advocacy groups for AYAs in Africa

Advocacy groups provide crucial support services, educational resources and a platform for collective action. These organisations empower AYAs to raise their voices and make a difference in their own healthcare journeys.

## Africa Cancer Foundation (ACF)

The ACF promotes cancer prevention and holistic solutions for those affected, with a focus on establishing best practices and advocating for increased government investment in cancer care [2]. Founded in Kenya, ACF works towards a cancer-free Africa by promoting prevention and providing holistic solutions for those affected by cancer, with a particular focus on AYAs. ACF is a trusted provider of cancer screening services in Kenya, and they advocate for governments to prioritise cancer control efforts and increase access to preventive, diagnostic and treatment services across the continent. The ACF understands the power of collaboration in tackling the complex challenge of cancer. Their best practice lies in forging strategic partnerships across diverse sectors. ACF has established successful collaborations with universities, faith-based organisations, insurance agencies, manufacturing businesses, technology companies and volunteer groups. These partnerships allow ACF to leverage the unique strengths of each entity, maximising their impact in reaching young adults with cancer across Africa. By working with governments on cancer initiatives, ACF further amplifies its advocacy efforts, ensuring a more comprehensive approach to improving access to prevention, screening and treatment for AYAs. This collaborative spirit exemplifies a robust best practice in cancer advocacy, paving the way for a future where no young African battling cancer faces the fight alone.

## The Cancer Association of South Africa (CANSA)

CANSA serves as a powerful model for advocacy efforts across the continent. This non-profit organisation champions the cause of all people affected by cancer, including AYAs. CANSA’s advocacy team relentlessly pushes for policy changes that ensure South African policymakers prioritise crucial cancer control issues [12]. Their unwavering commitment protects patients’ right to healthcare, guaranteeing access to essential services. CANSA recognises the power of information and community. They connect AYAs with vital community resources, fostering a sense of belonging and support. From educational materials to emotional counseling, CANSA provides a holistic approach to empowering AYAs facing cancer in South Africa. Their dedication serves as a beacon of hope, inspiring similar advocacy efforts across Africa to improve the lives of young people battling this disease.

## Childhood Cancer Foundation, South Africa (CHOC)

Founded in 1979, Childhood Cancer Foundation (originally known as Childhood Haematology Oncology Clinic) understands the importance of comprehensive support for children with cancer. They provide vital information and awareness programs to equip young people and their families with the knowledge needed for early diagnosis and treatment. Recognising the emotional and logistical challenges faced by families traveling long distances for childhood cancer treatment, CHOC provides a unique best practice: 13 ‘home-away-from-home’ facilities located near specialised treatment centers across South Africa. These no-cost accommodations offer children and a parent/caregiver a safe haven during treatment. CHOC goes beyond just a roof; they provide nutritious meals, a supportive environment with caring staff and opportunities for families to connect with others facing similar journeys [15]. This fosters a sense of normalcy and community, allowing families to focus on their child’s well-being while enjoying precious moments together outside the sterile hospital environment.

## Living with cancer, South Africa

This online platform empowers young people with cancer by creating a space to share their journeys, connect with peers and access valuable resources. Living with cancer recognises the power of storytelling and community. Through real-time member interaction and authentic experience sharing, AYAs can navigate their cancer journey with a sense of belonging and support [44]. Living with cancer, South Africa, recognises the power of shared experiences in navigating a cancer diagnosis. They champion a unique best practice: a virtual ‘Legacy of Love Memorial Wall.’ This online platform fosters a supportive community by allowing individuals to share stories, memories and reflections of loved ones lost to cancer. Through blog submissions, participants create a digital sanctuary, preserving the legacies of those who fought bravely. This honors loved ones and provides solace and inspiration to others facing similar challenges. By fostering connections and open dialogue, living with cancer goes beyond traditional support structures, creating a space for resilience and compassion in the face of adversity.

## Love your nuts South Africa (LYNSA)

LYNSA, a registered non-profit, tackles the sensitive issue of testicular cancer in young men. LYNSA tackles the critical issue of testicular cancer awareness, a topic often shrouded in silence, particularly among young men. LYNSA offers support networks for those diagnosed with testicular cancer, leveraging their network of survivors across Africa to provide ongoing guidance and encouragement throughout the treatment process. LYNSA, dismantles the cultural barriers surrounding testicular cancer through a unique best practice: the ‘Cancer Smart App.’ This innovative mobile application targets young men by providing them with accessible and age-appropriate cancer education. The app aims to empower young adults to take charge of their health by equipping them with the knowledge to perform self-examinations and recognise potential warning signs of testicular cancer [45]. By tackling sensitive topics head-on in a user-friendly format, Love Your Nuts breaks down cultural taboos and empowers young men to prioritise their health and seek early detection.

## Care for Cancer Foundation

The Care for Cancer Foundation has multiple locations in Africa. This foundation strives to increase access to essential cancer care and support for all, regardless of background. They play a crucial role in advocating for AYAs with cancer by creating pathways to access vital medical diagnostics in oncology [13]. Care for Cancer Foundation fosters partnerships with healthcare professionals and industry leaders to expedite diagnoses and treatment, ensuring a dignified and hopeful journey for young patients. They also offer free diagnostic procedures to underprivileged patients, promoting early detection and faster intervention. The Care for Cancer Foundation confronts the financial barriers to early detection faced by many African young adults through a powerful best practice: free medical diagnostics. They understand that a timely diagnosis is crucial for successful treatment. Care for Cancer Foundation removes a significant obstacle by providing free diagnostic procedures to underprivileged patients, enabling young adults to access essential testing regardless of their financial background. This not only improves access to care but also increases the chances of early detection, ultimately leading to better treatment outcomes and a brighter future for young African people battling cancer.

## Atinuke Cancer Foundation

Established in Lagos, Nigeria, the Atinuke Cancer Foundation is a powerful force for advocacy. Founded by a cancer survivor, Atinuke Cancer Foundation understands the unique challenges faced by AYAs diagnosed with cancer. They offer free cancer screenings, a crucial step in early detection, which is often a barrier for young people [76]. The Atinuke Cancer Foundation goes beyond screenings by providing educational programs specifically tailored for young adults. These programs equip AYAs with the knowledge they need to navigate their healthcare journey, fostering informed decision-making and a sense of empowerment. Atinuke Cancer Foundation does not stop at education; they also champion advocacy initiatives. The foundation, by raising awareness and advocating for policy changes, strives to create a healthcare system that prioritises the needs of AYAs with cancer in Africa. Their mission extends beyond individual patients; they aim to empower survivors, inspire hope and ultimately save lives through education and advocacy.

## Society of Oncology and Cancer Research in Nigeria (SOCRON)

The SOCRON is critical in advocating for public policy changes. They champion policies that guarantee access to high-quality cancer care for AYAs and actively support the development of clinical cancer research. Their dedication to inclusivity ensures that the needs of young people are represented in crucial conversations about cancer care and research priorities. Their dedication to inclusivity ensures that the needs of young people are represented in crucial conversations about cancer care and research priorities in Africa. The SOCRON champions a best practice crucial for improving AYA cancer care: evidence-based advocacy. Recognising the resource limitations in the Nigerian healthcare system, SOCRON goes beyond simply advocating for change. They partnered with leading organisations like American Society of Clinical Oncology to conduct research and develop resource-appropriate guidelines specifically tailored to the Nigerian context [72]. This ensures their advocacy efforts are grounded in scientific evidence and practical considerations, maximising their impact on policy changes directly benefiting AYAs with cancer in Africa. This data-driven approach fosters efficient resource allocation and the implementation of best practices within the existing healthcare infrastructure.

## The Dorcas Cancer Foundation

The Dorcas Cancer Foundation, a national non-profit organisation, recognises the devastating impact of childhood cancer on young Nigerians and their families. Their mission is clear: to ensure early detection, accurate diagnosis and prompt treatment for all childhood cancers. They achieve this through a multi-pronged approach, prioritising community awareness and health worker involvement. Educating communities about the signs and symptoms of childhood cancer empowers families to seek early diagnosis, a critical factor in successful treatment. Furthermore, The Dorcas Cancer Foundation collaborates with healthcare professionals, ensuring they have the knowledge and resources to diagnose childhood cancers accurately. However, their support does not end there.

The Dorcas Cancer Foundation tackles young Nigerians’ multifaceted challenges with childhood cancer through a powerful best practice: the Pediatric Cancer Access Program (PCAP) [23]. This program recognises that successful treatment goes beyond just medication. PCAP offers a holistic approach, ensuring completely free and uninterrupted care for children from diagnosis through all stages of treatment, including surgery, chemotherapy and radiation therapy. The Dorcas Cancer Foundation goes the extra mile by partnering with healthcare providers, institutions, pharmaceutical companies and private industry. These partnerships guarantee access to vital resources such as prosthetics and rehabilitation services, crucial elements of a child’s recovery journey. By addressing the financial and practical hurdles faced by families, PCAP empowers young Nigerians with a fighting chance against childhood cancer. Ultimately, The Dorcas Cancer Foundation serves as a beacon of hope for young Nigerians and their families facing childhood cancer, offering a comprehensive approach to support, treatment and a brighter future.

### Why age matters: the unique needs of AYA cancer advocacy

AYA is a crucial developmental stage marked by physical, emotional and social changes. A cancer diagnosis and treatment can significantly disrupt the unique developmental stage of adolescence and young adulthood, characterised by the exploration of identity, independence and the formation of intimate relationships. This disruption can lead to a sense of social isolation and a feeling of being ‘out of sync’ with their peers.

Handling a cancer diagnosis at such a young age might be extra challenging, as cognitive capabilities and emotional maturation are not fully developed, especially among younger AYAs [66]. A cancer diagnosis disrupts this delicate journey, presenting unique challenges for AYAs in Africa. Their needs often differ significantly from both children and adults with cancer. Compared with their ensured counterparts, AYAs with cancer are more likely to present with advanced disease and have poor prognoses [67]. While facing the physical fight against the disease, AYAs navigate a complex landscape shaped by their unique developmental stage. They are no longer children but not quite adults, often grappling with issues of identity, independence and future aspirations. This intersection of adolescence, young adulthood and cancer necessitates a distinct approach to advocacy that recognises the specific needs and challenges faced by AYA patients in Africa.

### Navigating the intersection of adolescence, young adulthood and cancer

The AYA years are a period of immense physical, emotional and social development. It is crucial to provide optimal care for AYAs with cancer in this delicate phase, as health professionals should engage multidisciplinary teams that offer physical and occupational therapy, nutrition and psychosocial support, along with medical expertise in tailoring cancer-directed therapy and symptom management [14]. AYAs in Africa are often completing their education, establishing independence and exploring romantic relationships. A cancer diagnosis disrupts this crucial developmental trajectory. The healthcare system often caters to distinct pediatric and adult oncology wards, leaving AYAs feeling like they do not quite belong in either. For AYAs, the diagnosis of cancer can negatively affect social development, especially with respect to education, employment and financial independence [71]. Treatment schedules may clash with school commitments, jeopardising educational opportunities.

The physical side effects of treatment can lead to social isolation and a sense of alienation from peers. During treatment, cancer-related fatigue negatively affects the lives of childhood, adolescent and young adult cancer survivors [16]. Furthermore, the emotional toll of cancer can be particularly devastating for AYAs, who may struggle to express their fears and anxieties openly. Long-term survivors of AYA cancer have higher rates of cognitive dysfunction and psychological distress as compared to the general population, due to the negative impacts of diagnosis and treatment [22]. The desire for independence often clashes with the reality of needing parental support, creating a complex dynamic within families. AYA cancer survivors have a high rate and number of physical, psychosocial and practical concerns developed during the treatment phase [38]. Effective advocacy for AYAs in Africa must acknowledge this complex interplay of developmental stages and cancer, ensuring their voices are heard and their specific needs are addressed throughout their healthcare journey.

## Specific barriers to advocacy for AYAs with cancer in Africa

Advocating for AYAs with cancer in Africa faces several significant barriers. These obstacles can be categorised into systemic, cultural, economic and personal challenges.

### Systemic barriers

Systemic barriers create significant challenges for AYAs with cancer in Africa. These barriers extend beyond diagnosis and treatment, often manifesting most acutely after treatment completion. A lack of access to holistic, multidisciplinary survivorship care leaves AYAs vulnerable [31]. Human resource shortages, coupled with geopolitical instability and quality management issues, further complicate access to quality care [47]. These systemic challenges are compounded by limited public awareness campaigns and socioeconomic disparities in health insurance coverage, creating a complex web of obstacles that AYAs with cancer must navigate. Others include:

- Healthcare infrastructure: Many African countries have underdeveloped healthcare systems with limited facilities, equipment and specialised cancer care centers. This is particularly acute in rural and underserved areas, where centralised medical services and infrastructure often lack sufficient resources.
- Policy and legislation: There is often a lack of policies specifically addressing the needs of AYAs with cancer. Existing healthcare policies may not adequately support cancer treatment and advocacy, leaving gaps in care and support.
- Workforce shortages: A scarcity of trained healthcare professionals, including oncologists, nurses and counselors who are crucial for cancer care and advocacy, exacerbates the challenges. This human resource shortage is often more pronounced in adolescent oncology.

### Economic barriers

The economic burden of cancer care presents a significant barrier for AYAs across Africa. These barriers include:

- Socioeconomic disparities and cost of treatment: Cancer treatment is prohibitively expensive for many families, with high costs associated with chemotherapy, radiotherapy and surgery. This burden is compounded by significant indirect costs such as transportation to medical facilities, accommodation during treatment and potential loss of income due to caregiving responsibilities. The situation is particularly acute for those living in rural or remote areas with limited income, creating a cycle of vulnerability [27]. These socioeconomic factors often lead to reduced utilisation of essential cancer care services, ultimately impacting treatment outcomes. As a result, AYAs may be forced into difficult choices, potentially delaying or forgoing necessary treatment due to the associated direct and indirect costs [10].
- Insurance coverage: Limited access to health insurance means that many patients have to pay out-of-pocket for their treatment, which is often unsustainable. This lack of financial protection can have a devastating impact on an AYA’s fight against cancer and their family’s economic stability.
- Funding for advocacy: There is a lack of funding for advocacy programs and initiatives aimed at supporting AYAs with cancer. The shortage of resources hampers efforts to raise awareness, provide support services and lobby for policy changes.

### Cultural barriers

In small African towns where cultural practices and beliefs hold strong influence, AYAs with cancer face significant barriers to engaging in advocacy activities. These young individuals, already burdened by their illness, must navigate a landscape where traditional values and societal norms often work against their desire to speak out and effect change.

- Stigma and misconceptions: Cancer is often stigmatised, and there are numerous misconceptions about the disease, which can lead to social isolation and reluctance to seek treatment. Misconceptions and myths surrounding cancer can delay diagnosis and treatment, hindering the effectiveness of care [3]. The stigma surrounding the disease means that even mentioning it can invite judgment and ostracism [50].
- Traditional beliefs and practices: In some communities, traditional beliefs and practices may take precedence over modern medical treatments, leading to delays in seeking appropriate care. These cultural barriers can lead to feelings of shame, social stigma and a reluctance to seek medical help or participate in advocacy activities [51].
- Sociocultural norms and taboos: In some African societies, respect for elders and strict hierarchical structures mean that young people are expected to remain silent and deferential [68]. AYAs with cancer who have often heard their parents and community leaders dismiss the voices of the youth, believing that matters of health and advocacy are not their concern [80]. This cultural norm stifles their desire to become an active advocate for better cancer care and support. These cultural realities can create an environment where there is a lack of open dialogue about cancer, hindering advocacy efforts. To address these challenges, nationwide public education campaigns that promote accurate information about cancer are crucial. Dispelling myths and fostering open dialogue with AYAs can empower them to prioritise their health, seek timely medical intervention and engage in meaningful advocacy.
- Communication gaps: Differences in language and literacy levels can hinder effective communication between healthcare providers and patients, making advocacy more challenging.

### Personal barriers

A cancer diagnosis has a great impact on the already complex developmental journey of AYAs in African countries. This creates unique personal barriers hindering effective advocacy [60]. Advocacy efforts must address these personal barriers by fostering open communication, providing age-appropriate information and building strong support networks for AYAs battling cancer in Africa.

- Psychosocial support: The emotional and social turmoil caused by cancer can lead to a reluctance to accept social support, hindering their ability to cope with the challenges of the disease [8]. AYAs with cancer require specialised psychosocial support, which is often lacking [31]. This can include counseling, peer support groups and mental health services.
- Awareness and education: A lack of awareness about cancer and its treatment options can leave AYAs feeling powerless and uninformed. There is a general lack of awareness among AYAs and their families about cancer symptoms, treatment options and the importance of early detection. Understanding the specific communication and informational needs of AYAs is crucial to bridging this gap and empowering them to participate actively in their own care [8].
- Empowerment: AYAs may feel disempowered or lack the confidence to advocate for their needs due to their age and the nature of their illness. This can be exacerbated by the emotional and physical challenges of cancer treatment.

## Discussion

### Addressing specific challenges faced by AYAs with cancer

AYA cancer patients in Africa face a multitude of challenges beyond the immediate fight against the disease. A cancer diagnosis during the AYA years is a traumatic event, and the impact leads to the manifestation of psychiatric disorders during the AYA period and long-term mental health outcomes [19]. Limited access to specialised care tailored for their age group can create significant barriers to optimal treatment outcomes. Furthermore, a lack of awareness and understanding of AYA cancers among healthcare professionals can lead to misdiagnosis or delayed diagnosis. Cancer and its treatment can result in lifelong medical financial hardship and survivors of AYA cancers are more likely to experience medical financial hardship compared with adults without a cancer history [46]. The financial burden of treatment can be particularly acute for young adults, who may not yet have established financial independence. AYAs with cancer are particularly vulnerable to the rising cost of cancer, which represents a stressor that has significant and lasting effects on quality of life [69].

Greater attention is needed to address AYA-specific concerns on independence, emotional well-being and social support [60]. When addressing the specific challenges faced by AYAs with cancer, it is crucial to acknowledge the significant impact that the disease can have on their social relationships and overall social functioning [52]. A review of the literature has highlighted that there has been limited focus on this aspect, despite the fact that AYAs with cancer often face greater challenges in maintaining and developing social relationships compared to their peers without cancer [78]. Several factors contribute to the social difficulties experienced by AYAs with cancer. The physical and emotional toll of the disease, coupled with the demanding treatment regimens, can make it challenging for AYAs to participate in typical social activities and maintain connections with their peers. Additionally, changes in physical appearance, such as hair loss or weight fluctuations, can lead to feelings of self-consciousness and a reluctance to engage in social situations. It is essential to recognise the importance of social support and relationships for AYAs with cancer. Positive social connections have been shown to contribute to better psychological well-being, adherence to treatment and overall quality of life. Interventions and support services should be designed to address these social challenges and facilitate the maintenance and development of meaningful social relationships for AYAs with cancer.

Additionally, the social stigma surrounding cancer can be particularly isolating for AYAs in Africa, where cultural beliefs may lead to feelings of shame and discrimination. Developing age‐appropriate late‐effect communication strategies that recognise high AYA distress may help address the gap between desired information and perceived information quality [26]. Effective advocacy for AYAs in Africa must address these specific challenges. This includes advocating for increased access to age-appropriate oncology services, raising awareness among healthcare professionals and implementing financial support systems to alleviate the burden on families. Furthermore, advocacy efforts can challenge social stigma and promote open conversations about AYA cancers within communities.

Being an AYA with cancer increases the risk for future recurrence and exposure to new cancers throughout one’s life [37]. This heightened risk is particularly pronounced for African AYAs with cancer, who face an even greater susceptibility to developing secondary cancers and experiencing recurrences [30]. The increased risk for African AYAs can be attributed to a combination of factors: First, there may be disparities in access to cancer screening, early detection and high-quality treatment for African AYAs, which can lead to more advanced stages of cancer at diagnosis and suboptimal treatment outcomes, increasing the likelihood of recurrence or the development of new cancers. Second, some African AYAs may face certain risk factors that could potentially increase their risk for future cancers. While the prevalence of these risk factors can vary widely across different African countries and populations, it is important to consider potential contributors. For example, obesity has been identified as a risk factor for certain cancers. Specifically, a significant association has been found between obesity and leukemogenesis in the development of Childhood and AYA acute lymphoblastic leukemia [25]. Other factors, such as physical inactivity and exposure to environmental carcinogens, may also further compound their risk for future cancers. Additionally, there may be genetic and biological factors specific to African populations that contribute to an elevated risk for certain types of cancers or an increased susceptibility to the long-term effects of cancer treatments. For example, BRCA1 and BRCA2 mutations are known to increase the risk of breast and ovarian cancers. Some studies suggest that certain mutations in these genes may be more prevalent in African populations [9,36,56]. Also, the tumor suppressor gene, TP53, is often mutated in various cancers, including breast cancer, particularly in African populations. Likewise, APC Mutations, associated with colorectal cancer, can be more frequent in African populations [77]. A seminal work by Huo *et al* [33] on the over-representation of triple-negative breast cancer (TNBC) highlights its prevalence among African and African American women. The study by Huo *et al* [33] revealed that TNBC is more common in these populations, potentially due to genetic and environmental factors. The research found that this subtype of breast cancer lacks estrogen, progesterone and HER2 receptors, making it more challenging to treat and often resulting in a poorer prognosis compared to other breast cancer types. The consequences of future cancer recurrence or the development of new cancers can be devastating for all AYAs, but African AYAs may face additional challenges due to socioeconomic barriers, cultural factors and potential disparities in access to follow-up care and support services. By acknowledging the heightened risk for African AYAs with cancer and implementing targeted, culturally sensitive interventions, healthcare providers, policymakers and support systems can work towards reducing disparities and improving the long-term outcomes and quality of life for this vulnerable population. Research efforts aimed at the survivorship care of this unique population are needed [37].

Poverty and financial constraints significantly limit AYAs with cancer from participating in advocacy initiatives [18]. Financial instability often means these individuals must prioritise their immediate medical and living expenses over other activities. The costs associated with cancer treatment, such as chemotherapy, radiation, medications and frequent medical appointments, can be overwhelming, leaving little to no room for additional expenses related to advocacy work. Moreover, many AYAs with cancer face challenges in maintaining employment or continuing their education, further exacerbating their financial difficulties. The loss of income or educational opportunities can lead to increased stress and a focus on survival rather than advocacy. Without the necessary financial support, AYAs may struggle to afford travel, accommodation or even basic participation fees for advocacy events, conferences or workshops.

Additionally, the time and energy required for cancer treatment and recovery can be immense, often leaving AYAs physically and emotionally drained. This exhaustion can make it difficult for them to engage actively in advocacy efforts, which typically require a considerable commitment of time, energy and resources [52]. The need to prioritise health and well-being over advocacy is a reality for many AYAs facing financial hardship. Furthermore, the psychological impact of living in poverty can diminish self-confidence and the perceived ability to effect change, discouraging AYAs from becoming involved in advocacy initiatives. Consequently, the stress and anxiety associated with financial constraints can overshadow the motivation and drive needed to participate in or lead advocacy efforts.

### Recommended strategies to overcome these barriers

Based on the analysis of the specific barriers to advocacy for AYAs with cancer in Africa, the following recommendations are hereby made (Table 1).

## Conclusion

Based on existing literature, expert opinions and case studies, this paper explores several major barriers, including the social and cultural stigma around cancer, limited access to health education and support services, economic difficulties and insufficient healthcare infrastructure. Sociocultural norms and taboos can prevent open conversations about cancer, hampering advocacy efforts. Moreover, poverty and financial challenges often limit access to treatment and support resources, reducing opportunities for AYAs to participate in advocacy initiatives. The inadequacy of healthcare systems, lack of specialised care facilities and limited training for healthcare professionals also pose significant obstacles. This paper underscores the urgent need to tackle these multifaceted barriers through joint efforts involving healthcare providers, policymakers and community organisations. Addressing these barriers requires a multifaceted approach involving collaboration between governments, healthcare providers, non-governmental organisations, communities and international partners.

## List of abbreviations

ACF, Africa Cancer Foundation; AYA, Adolescents and young adults; CANSA, Cancer Association of South Africa; CHOC, Childhood Cancer Foundation, South Africa; LYNSA, Love Your Nuts South Africa; PCAP, Pediatric Cancer Access Program; SOCRON, Society of Oncology and Cancer Research in Nigeria.

## Conflicts of interest

The authors declare no conflicts of interest.

## Funding

The authors received no financial support for the research, authorship and/or publication of this article.

## Author contributions

- CUN: Conceptualisation, Methodology, Writing – Original Draft Preparation, Supervision, Project Administration. CUN led the development of the manuscript, designed the review methodology and provided guidance throughout the project.
- OS: Investigation, Data Curation, Writing – Review and editing. OS contributed to the collection and synthesis of relevant literature, critically revised the manuscript and provided intellectual input on key sections of the review.
- AFO: Visualisation, Writing – Review and editing, Formal Analysis. AFO was responsible for visualising the findings, reviewing the data analysis and contributing to the final editing of the manuscript.
- MOO: Conceptualisation, Methodology, Data collection, Writing – original and revised draft preparation, manuscript revision.

## Figures and Tables

**Table 1.** Recommendations.

Recommendations	Description
Strengthening Healthcare Systems in Africa	This includes investing in healthcare infrastructure, training healthcare professionals, and developing specialized cancer care centers to improve access to care for AYAs with cancer.
Advocating Policies for AYA Cancer Care	Cancer care professionals must continue to advocate for the creation and implementation of policies that specifically address the needs of AYAs with cancer.
Boosting Funding for Cancer Treatment and Advocacy through Multi-Sector Support	Increasing funding for cancer treatment and advocacy programs through government support, international aid, and private-sector partnerships.
Public education campaigns	Conducting public education campaigns to dispel myths about cancer, reduce stigma, and promote early detection.
Strengthening support systems	Developing comprehensive support systems that include counseling, support groups, and mental health services tailored to AYAs.
Blending Traditional and Modern Medicine with Culturally Sensitive Advocacy	Integrating traditional and modern medicine approaches and ensuring that advocacy efforts are culturally sensitive and community-driven.
Peer support programs	Connecting AYAs with cancer with others who have gone through similar experiences can provide a sense of understanding and validation, as well as opportunities for social interaction.
Social skills training	Offering programs that help AYAs develop or strengthen their social skills, such as communication, assertiveness, and conflict resolution, can aid in navigating social situations more effectively.
Facilitated social activities	Organizing age-appropriate social events and activities specifically for AYAs with cancer can create opportunities for socialization and the formation of supportive relationships.
Regular follow-up care	Ensuring consistent follow-up care with healthcare providers and adherence to recommended cancer screening guidelines
Addressing sociocultural barriers	There must be targeted efforts to address socioeconomic and cultural barriers that may impede access to preventive care and support services.
